# Editorial

**Published:** 1915-05

**Authors:** 


### EDITORIAL

The Business Office of the *Journal-Record* is 23 West Alabama Street.

The Editorial Office is 1014-15 Atlanta National Bank Building.

Address all Business Communications to *Journal-Record of Medicine*, 23 West Alabama Street.

Make remittances payable to *The Journal-Record of Medicine*.

On matters pertaining to the Editorial and Original Communications, address Edgar G. Ballenger, M. D., Atlanta, Ga.

Reprints of Original Articles will be furnished at cost price. Requests for the same should always be made in THE MANUSCRIPT.

We will present, postpaid, on request to each contributor of an original article, twenty (20) marked copies of *The Journal-Record of Medicine* containing such article.

### CHANGE OF EDITORS

Owing to his professional demands, Dr. Edgar G. Ballenger has resigned his position as Editor of the Journal- Record of Medicine. His resignation will not affect the policy of the Journal, as Dr. R. R. Daly, who has assisted with the editorial work for several years, will assume the editorship.

---

### THE TAXATION OF PHYSICIANS.

Under the present Georgia law, physicians are taxed ten dollars yearly for the privilege of practicing medicine within the borders of the State.

This is not a large amount and in the majority of instances it cannot be shown to be a great burden for the doctor to carry. None the less it is irksome and for very good reasons. In the first place the quid pro quo is lacking. It is contrary to our spirit of fair play to give something for nothing no matter if receiving something for nothing happens to be the ruling ambition of many people and communities. The physician in Georgia is no better off and he has no more protection of the laws than any other citizen who pays no special tax. It may be said that he is exempt from jury duty, but the reason for that apparent privilege lies in the imperative need the people frequently have for his services when they would brook no delay that even the sacred customs of the court might impose upon his answering the summons to the bedside. It is not his privilege but that of the people he attends—this freedom from jury duty. Besides, the thoughtful doctor is accustomed to seeing thinsg as they are and to making up his mind entirely independently of all sophistry and specious argument. The rules of evidence are of no moment beside the evidence itself and he is taught by the immanence of death to recognize evidence when he sees it. Thus, his training rather unfits him for the peculiar mental condition the juryman is supposed to make himself occupy.

There are other classes that are free from jury duty and some of them pay special taxes and others do not.

The special tax payers seem to be lawyers, doctors, stud horses and jackasses. The doctors certainly keep away from juries as far as possible and they desire to get entirely away from the special tax group, however congenial the other members may find the association.

On the grounds then of unfair taxation because nothing is given in return; of more or less burdensome expense; of enforced uncongenial association, the Legislature will be asked to change the tax laws so as to relieve the doctors.

Every physician knows what he does not often say that is that the amount of effort he puts forth in unpaid work for the indigent of the community is a large percentage of his daily labor. He knows, too, that often enough his services are engaged by the state and county when there will be no recompence that will buy bread and butter. He responds to these demands cheerfully because it is part of his humanity and of his training to do so, but when he has returned to his interrupted meal or his disturbed rest after such a visit and finds a tax bill or notice awaiting him, he surely resents it.

If every doctor in Georgia will let his legislator know his feelings upon this matter within the next month, the tax will disappear at the summer session. R. R. D.

### WAR BABIES.

A newspaper carried the statement that the priests in Belgium were commending infanticide to such women as found themselves likely to become mothers of children begotten of invading and alien fathers, and later on it printed an authoritative denial of the priests giving such advice.

It was not denied that there were such mothers and the inference is that there are enough of them to make it more than an individual question.

It has always been the custom of invaders to seize the conquered country and to take from it what they wanted and could find. Always, that is, until the last century when we began to attach real meaning to such terms as civilized and barbarous and to set ourselves and our ethics proudly and determinedly in the civilized class. Women were the spoil of war in the ancient times as described in sacred and profane writings, and many penalties were exacted or privileges accorded to the men concerning them. It was all "barbarous" to us and very distant until this war has brought things home.

Is our vaunted civilization only skin deep? Would we, ourselves, degenerate under the stress of murderous warfare to common rapists and wanton destroyers? Did we, when at each other's throats during the civil war commit acts that would cause us shame when we viewed them by our peacefu lstandards? Destruction was everywhere in those struggling and fighting days and homes were burned or occupied by the fighters when fighting seemed to demand it, but who can point to raping and to war babies as a result? Women were as sacred to the soldiers as to the men, and our romanic tales of that war turn very often upon the heroism of the women left to guard homes successfully. More than that the love tales of men who invaded the land and became so enamoured of the women that they returned to marry them, have so much of truth that their verisimilitude is the unquestioned theme of picture shows today.

No, we are not rapists any more than we are the stolid and uncomplaining followers of vicious warlords who destroy or burden the land. We are different and our women are different. Our civilization is more than skin deep on that question and we do not have to parade it as "kultur" or any other special name to be guided by its influence, no matter what the stress.

Thus it is next to impossible for us to judge the situation or to understand the feelings of the people where injuries have been suffered during the last ten months. The Belgians are rae orderly, home-loving, alert and peaceful people. They have had the respect of the world for generations because of their racial independence and the struggles they have made to retain it. Their country has been the battlefield of Europe, but they have lived and fought through it to the proud position of having won without claiming the world's respect. When a nation has done this, where rests its greatest pride? The women of the land are its real patriots; they are the conservers of traditions for it is with these that they are to build heroes in their sons. It is the mother's pride in country that looks upon the land as the place for the glory of her son.

In some such way as this one may arrive at the viewpoint of the woman carrying an unwelcome child of an enemy. Shall she let it live? Can she sacrifice it because of the hateful circumstances of its conception, or is the sense of possession and of motherhood great enough to isolate the father and cast him off so that the babe may remain? Mother love is the unlearned power of the world. It sacrifices self and suffers pain. It carries depression to the lowest depths in the hope of happiness for the child. It fights openly and hiddenly, fairly and unfairly, for the tenderest of things. It strives unremittingly after what is patently unattainable and finds joy in the strife; yet sometimes it kills. The newspapers have headings of "Mother Strangling Two Babies and then Killing Herself." Such like things come often enough to tell us that she can and will kill under certain provocations.

Should the child's lineaments resemble those of the man she never more would see and whom she will always hate, would that be the insurmountable obstacle to mother love and care? She might feel a certain victory in raising a half German son to hate Germany and to be a loyal Belgian and this might help her to set aside the ideals of her life for her chosen children, but could she stand the picture, the constant reminder of the brutal enemy that assaulted her virtue and destroyed her aspirations?

These are some of the questions to be answered very soon and it may be that the annexation of Belgium is part of the reply. But beyond it all there is the real feminine point of view which no man can reach or occupy or understand. It is elusive, tantalizingly so, and no woman can tell about it were she so disposed.

The effects of the war when it is over will be measured in part by this element and it is a problem for the psychologists to note. R. R. D.

